# Dynamic regulation of semaphorin 7A and adhesion receptors in ovarian follicle remodeling and ovulation

**DOI:** 10.3389/fcell.2023.1261038

**Published:** 2023-10-24

**Authors:** Alaknanda Emery, Kylie R. Dunning, Doan T. Dinh, Lisa K. Akison, Rebecca L. Robker, Darryl L. Russell

**Affiliations:** ^1^ The Robinson Research Institute, School of Biomedicine, University of Adelaide, Adelaide, SA, Australia; ^2^ School of Biomedical Sciences, University of Queensland, Brisbane, QLD, Australia

**Keywords:** granulosa cells, ovulation, adhesion, semaphorin, plexin, integrin, cumulus-oocyte complex

## Abstract

The ovarian follicle is a complex structure that protects and helps in the maturation of the oocyte, and then releases it through the controlled molecular and structural remodeling process of ovulation. The progesterone receptor (PGR) has been shown to be essential in regulating ovulation-related gene expression changes. In this study, we found disrupted expression of the cellular adhesion receptor gene *Sema7A* in the granulosa cells of PGR^−/−^ mice during ovulation. We subsequently found that expression of *Sema7A* in preovulatory follicles is promoted by gonadotropins and hypoxia, establishing an asymmetrical pattern with the SEMA7A protein enriched at the apex of large antral follicles. *Sema7A* expression was downregulated through a PGR-dependent mechanism in the periovulatory period, the abundance of SEMA7A protein was reduced, and the asymmetric pattern became more homogeneous after an ovulatory stimulus. Receptors for Sema7A can either repel or promote intercellular adhesion. During ovulation, striking inverse regulation of repulsive Plxnc1 and adhesive Itga5/Itgb1 receptors likely contributes to dramatic tissue remodeling. The adhesive receptor Itga5 was significantly increased in periovulatory granulosa cells and cumulus–oocyte complexes (COCs), and functional assays showed that periovulatory granulosa cells and COCs acquire increased adhesive phenotypes, while Sema7A repels granulosa cell contact. These findings suggest that the regulation of Sema7A and its associated receptors, along with the modulation of integrin α5, may be critical in establishing the multilaminar ovarian follicle structure and facilitating the remodeling and apical release of the cumulus–oocyte complex during ovulation.

## Introduction

Mammalian oocytes develop within ovarian follicles, which are complex structures of several cell and extracellular matrix (ECM) layers. Once matured, these oocytes are selectively released from the follicle into the fallopian tube or oviduct, where fertilization occurs. Structural and molecular changes within the follicle initiated by the luteinizing hormone (LH) surge mediate oocyte release through a cascade of gene expression that mediates expansion of the cumulus–oocyte complex (COC) and oocyte maturation (Russell and Robker, 2007; Suebthawinkul et al., 2022), and reduced structural integrity of the follicle wall, leading to the release of COC through the follicle apex (Duffy et al., 2019). The specific molecular mechanisms necessary for ovulation are poorly understood, but ovarian expression of progesterone receptor (PGR) is known to play an obligatory role in regulating transcriptional changes in genes required for ovulation (Robker et al., 2000; Dinh et al., 2019; Dinh and Russell, 2022; Dinh et al., 2023). PGR is a nuclear receptor transcription factor that is acutely induced in granulosa cells responding to the LH surge, and PGR broadly regulates periovulatory gene expression changes. Ovulation failure occurs in mice with PGR null mutation (Robker et al., 2000) and in humans treated with PGR antagonists (Cahill et al., 2022), prompting the suggestion that PGR is a master transcriptional regulator of critical ovulation mediators and the effectors of this mechanism are actively sought.

It has been demonstrated that expanded preovulatory COCs adopt a robust capacity to adhere to ECM proteins and invasively migrate through barrier ECMs of collagen I, collagen IV, or fibronectin. This phenotype is induced by the LH surge, peaking at the time of ovulation and declining very rapidly after the release of COC (Akison et al., 2012). Collagens and fibronectin are abundant in the periovulatory follicle, as well as stroma and surrounding ECM (Tomaszewski et al., 2021), and these structural ECM proteins are mediators of cellular anchoring, motility, and invasive capacity in systems, such as wound healing and cancer metastasis (Dalton and Lemmon, 2021).

Semaphorins are a family of cell guidance proteins that modulate attraction or repulsion of cells on contact with proteins in the extracellular environment. Semaphorins interact with plexin cell surface receptors and other co-receptors, and regulate integrin-mediated cell migration [reviewed in Tamagnone and Comoglio (2004)]. Many cell types continually sense extracellular guidance cues, and migrating cells require these signals to support the progressive movement in a specific direction (attractive cues) or prevent further migration (repulsive cues) [reviewed in Giacobini and Prevot (2013)]. Semaphorin 7A is a cell surface protein that specifically interacts through cell contact with specific extracellular cues to guide cell movement and spatial patterning, and establish specialized tissue structures. A semaphorin 7A interaction with Plexin C1 causes the collapse of cytoskeletal actin filaments and repulsion of cell contacts, while a semaphorin 7A interaction with integrins α5β1 or α1β1 promotes cell attachment (Scott et al., 2009). The roles of semaphorins are best known in patterning cell migration to establish neuronal architecture (Jongbloets et al., 2013) or regulate the motility of cancer cells (Song et al., 2021), but their potential role in regulating ovarian function has not been explored.

To address the hypothesis that hormone-regulated expression of cellular guidance receptors mediates the patterning of follicle structures and ovulation, the present study investigated the expression of cell adhesion receptors in cells of the ovarian follicle during ovulation. The role of PGR in regulating these genes was determined in order to identify cellular adhesion/invasion pathways of particular importance for ovulation. Distinct hormonal regulation and spatial patterning of semaphorin 7A protein at key follicular stages, along with functional demonstration that semaphorin 7A is anti-adhesive to granulosa cells, supports in playing a role in establishing the ovarian follicle structure and facilitating remodeling and apical release of COC at ovulation.

## Materials and methods

### Materials

Equine chorionic gonadotropin (eCG) was purchased from the National Hormone and Peptide Program (NHPP) (Torrance, CA, United States), and human chorionic gonadotropin (hCG) was purchased from Schering-Plough Pty Ltd.. Culture medium was purchased from Gibco, Invitrogen, Australia Pty Ltd. All other reagents were purchased from Sigma-Aldrich Pty Ltd., unless otherwise stated.

### Animals

All mice were maintained at Laboratory Animal Services (Adelaide, SA, Australia) on a 12 h light:dark cycle, with room temperature between 18ºC and 24°C and humidity between 40% and 70%. The mice were housed under specific pathogen-free conditions in individually ventilated cages, and rodent chow (specialty feed) and water were provided *ad libitum*. All experiments were approved by the University of Adelaide’s ethics committee and were conducted in accordance with the National Health and Medical Research Council Australian Code for the Care and Use of Animals for Scientific Purposes.

CBA x C57BL/6 first filial generation (F1) (CBAF1) female mice were obtained from Laboratory Animal Services (University of Adelaide, SA, Australia). Female PRlacZ-knock-in mice were generously provided by Associate Professor John Lydon (Baylor College of Medicine, Houston, TX), and experimental mice were generated via an in-house breeding colony. LacZ insertion results in disrupted transcription of both isoforms of PGR (Robker et al., 2000) and are hereafter designated as PRKO. Mice heterozygous for lacZ insertion (PR+/−) exhibit normal fertility (Robker et al., 2009). At weaning, the offspring were genotyped by two-way PCR analysis of tail DNA using primers specific for the Pgr gene and the neomycin gene: Pgr sense 5′ TAG ACA GTG TCT TAG ACT CGT TGT TG 3′; Pgr antisense 5′ GAT GGG CAC ATG GAT GAA ATC 3′; and Neo 5′ CTT CAC CCA CCG GTA CCT TAC GCT TC 3′. The Neo primer was used with a primer from the Pgr gene to amplify a 110-bp product from the mutant allele. A 590-bp WT product was created using the two Pgr primers.

### Isolation of mouse COCs and granulosa cells

Prepubertal (21–23 days of age) female mice were treated with eCG (5 IU), followed by hCG (5 IU i.p.) 44 h later. Ovaries or oviducts were dissected from the mice, and COCs and granulosa cells were collected either prior to the eCG treatment; 44 h post-eCG; or 4, 8, 12, (preovulatory) or 16 h (postovulatory) post-hCG. Cumulus–oocyte complexes and granulosa cells were collected by a puncture of large antral follicles (preovulatory) or by a puncture of the oviductal ampulla (ovulated COCs; 16 h post-hCG). All cell samples were pooled from at least five animals for each biological replicate for the CBAF1 time course (*n* = 3–5 replicates per time point). PRKO, PR+/− COCs, and granulosa cells obtained from individual animals constituted the biological replicates to compare genotypes (*n* = 5 per genotype per time point). Cells required for RNA analysis were snap-frozen in liquid nitrogen and stored at −80°C until use or used fresh in adhesion assays. Preovulatory whole ovaries were collected via dissection, fixed in 4% paraformaldehyde, and embedded in paraffin for immunohistochemistry.

### Granulosa cell culture

For gene expression analysis, granulosa cells isolated from eCG-primed mice were centrifuged at 1,000 g, counted, and seeded at 2 × 10^4^ cells per well in 96-well culture plates previously coated with DMEM/F12 media containing 5% FCS. After overnight culture in standard incubation conditions, the cells were washed and the media was replaced with DMEM/F12 containing a vehicle or 50 mIU/mL of FSH. Separate plates were incubated in atmospheric oxygen (20%), 5% CO_2_, and 37°C, while the other samples were placed into a sealed chamber equilibrated with 2% oxygen and 5% CO_2_, and placed into the same incubator. After 2 h treatment in these conditions, the cells were harvested and processed for RNA extraction, as described.

For cell morphology analysis, cells were plated in wells pre-coated for 2 h with DMEM/F12 medium containing either 5% FCSor bSA, fibronectin, or semaphorin 7A at the indicated concentrations. After 2 h, the cells were washed with fresh serum-free media and imaged. For cytoskeletal imaging, the cells were fixed in 4% formaldehyde, washed in PBS, and then permeabilized with 0.1% Triton X-100 in PBS for 15 min. The cells were then incubated for 15 min in PBS containing 1X Phalloidin-Alexa-488 (Thermo Fisher) and 1% bSA, then washed in PBS containing Hoechst 33342, and imaged on an Olympus IX81 inverted fluorescent microscope.

### Gene expression analysis

Total RNA was isolated from frozen samples and reverse transcribed into cDNA, as previously described [3]. Briefly, cells were homogenized in TRIzol (Invitrogen, Life Technologies, Victoria, Australia), as per the manufacturer’s instructions, including 7.5 μg of GlycoBlue (Ambion, Life Technologies, Mulgrave, Victoria, Australia) during RNA precipitation at −20°C. Total RNA was then treated with 1 U of DNase (Ambion), and RNA concentration and purity were quantified using a NanoDrop ND-1000 spectrophotometer (BioLAB Ltd., Victoria, Australia). First-strand complementary DNA (cDNA) was synthesized from total RNA using random hexamer primers (GeneWorks, Hindmarsh, South Australia) and SuperScript III reverse transcriptase (Invitrogen, Life Technologies, Mulgrave, Victoria, Australia). Real-time PCR reaction controls included omission of the cDNA template or reverse transcription enzyme in otherwise complete reactions.

TaqMan Gene Expression assays were purchased from Applied Biosystems (Itga1 Mm01306375_m1, Itga5 Mm00439797_m1, PlxnC1 Mm01236713_m1, Rpl19 Mm02601633_g1, Sema7A Mm00441361_m1, and Vegfa Mm00437306_m1), and reactions were run in duplicate on an AB7900HT Fast PCR system using the manufacturer’s recommended amplification settings. Each reaction used 0.5 μl of TaqMan assay, 5 μl of TaqMan master mix, 5 ng of cDNA, and H_2_O adding up to a final volume of 10 μl. Ribosomal protein L19 (*Rpl19*) was used as the endogenous reference gene in the analysis of gene expression using the 2^−ΔΔCT^ method of quantification.

### Immunohistochemistry

Sections (5 μm) of paraffin-embedded tissues were mounted onto SuperFrost microscope slides (Thermo Scientific) and either used for immunofluorescence or diaminobenzidine (DAB) immunohistochemistry. Tissue sections were dewaxed, rehydrated, and blocked in 10% normal goat serum (1 h, RT), followed by incubation with primary antibody (semaphorin 7A, Abcam ab23578, or integrin α5 1:500, Merck MAB2575) or isotype-matched control IgG (Rabbit polyclonal IgG, 1:500) overnight at 4°C. Sections were then washed and incubated with secondary antibodies (biotinylated goat anti-rabbit 1:500, Millipore Corporation, or Alexa Fluor 594 goat anti-rabbit IgG 1:500, Life Technologies) for 1 h at RT. Immunolabeling was visualized through incubation with streptavidin horseradish peroxidase conjugate (Vectastain ABC Kit; Vector Laboratories), and detection was performed using DAB (Vector Laboratories) and counterstained with hematoxylin, prior to mounting. Images were captured at high resolution using the NanoZoomer Digital Pathology technology (Hamamatsu Photonics K.K.). For immunofluorescence, Hoechst 33342 (Life Technologies) was added with the secondary antibody (1:250); sections were then washed in TBS and mounted with fluorescence mounting medium (Dako), and then imaged by confocal microscopy (Leica SP5 Spectral Scanning Confocal Microscope).

### Real-time cell adhesion assays

The xCELLigence Real-Time Cell Analyzer (RTCA) DP instrument (Agilent) was used to measure adhesion of granulosa cells isolated from the follicles of mice at preovulatory (eCG 44 h) or periovulatory (eCG + hCG at indicated times) stages using the methods previously described (Akison et al., 2012). Briefly, E-plates were coated with pure recombinant mouse fibronectin 5 μg/mL (R&D Systems) at the indicated concentrations for a minimum of 4 h at 37°C/5% CO_2_. Granulosa cells (10,000 cells per well) were loaded into the coated wells, and cell impedance measurements were recorded at 2 min increments for 2 h, at which time the cell adhesion index was recorded. Each treatment was run in triplicate technical replicates and repeated 3–5 times in independent experimental replicates.

### Statistical analyses

Data shown are mean ± SEM across independent experiments for adhesion assays and real-time PCR. Experiments were analyzed using a one- or two-way analysis of variance (ANOVA), followed by the Holm–Sidak *post hoc* multiple comparison procedure to determine statistical differences between groups. Before any analysis, all data sets were tested for normality and equal variances using the Kolmogorov–Smirnov test. Where data were identified as not being normally distributed, a log transformation was performed. All analyses were performed using the SigmaPlot 12.5 statistical package (Systat Software Inc.).

## Results

### Dynamic regulation of ovarian *Sema7A* expression by hormones and hypoxia

**FIGURE 1:**
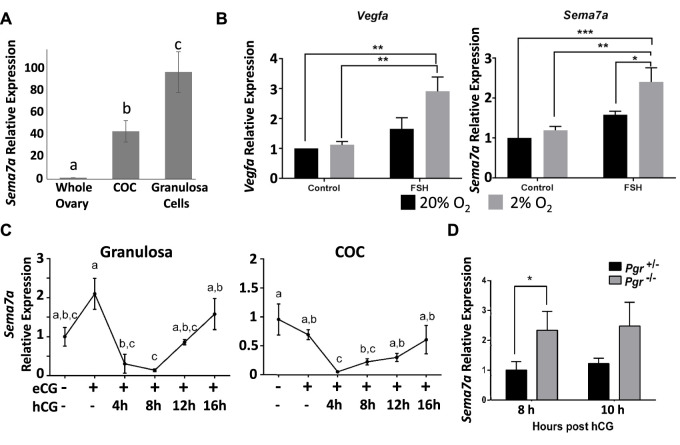
Expression of Sema7A in granulosa cells is induced through hypoxia and FSH signals and repressed via PGR after ovulatory stimulus. **(A)** Sema7A expression is abundant in COCs and granulosa cells. **(B)** In cultured granulosa cells, Sema7A and Vegfa are induced by FSH in a hypoxic environment. **(C)** Ovulatory hCG stimulus causes rapid downregulation of Sema7A expression in granulosa cells. **(D)** Elevated Sema7A expression in PGR^−/−^ compared to PGR^+/−^ periovulatory granulosa cells (hCG 8 or 10 h treatment).

A comparison of Sema7A in whole ovaries versus isolated granulosa cells or COCs at the preovulatory stage (21-day-old mice with no exogenous gonadotropin treatment) showed over 90-fold enrichment of *Sema7A* expression in granulosa cells and 40-fold in COCs compared to the whole ovary, illustrating that *Sema7A* is selectively expressed and likely plays a distinct role in the intrafollicular compartment (Figure 1A). In cultured granulosa cells, treatment with follicle-stimulating hormone (FSH) alone did not increase *Sema7A*, but co-stimulation with FSH and low oxygen tension (2% O_2_) caused a significant 2.5-fold increase in Sema7A mRNA abundance. Likewise, the FSH- and hypoxia-dependent gene *Vegfa* (Li et al., 2020) showed a similar pattern and magnitude of response (Figure 1B). Further analysis of granulosa cells and COCs isolated from *in vivo* eCG-stimulated follicles also showed a two-fold increase in *Sema7A* expression in granulosa cells but not in COCs, while induction of ovulation through hCG treatment caused a significant >10-fold reduction in *Sema7A* expression in granulosa cells and COCs after hCG ovulation stimulus (Figure 1C). Microarray and RNA-seq (Dinh et al., 2023) analyses of periovulatory (8 h post hCG) granulosa cells from an ovulatory PGR-null mice showed significantly higher *Sema7A* expression than WT controls, following hCG treatment (not shown), consistent with a reduced level of downregulation after ovulatory stimulus when PGR was deleted. This was further quantitatively measured to verify dysregulation of *Sema7A* expression by qPCR in granulosa cells of PRKO and PR−/+ mice after both 8 and 10 h of hCG treatment, times when the expression of PGR is highest (Dinh et al., 2019) and Sema7A is lowest in granulosa cells. *Sema7A* expression was significantly higher by 2.3-fold and 2-fold at 8 and 10 h post-hCG, respectively, in PRKO compared to the heterozygous littermates (Figure 1D).

Together, these findings indicate that Sema7A is highly expressed in granulosa and cumulus cells through the combined action of the folliculogenic hormone FSH and the hypoxic environment in the ovarian follicle, and is rapidly downregulated through a PGR-dependent mechanism in response to ovulatory stimulus. This PGR-mediated downregulation is a rare observation that sets the response to Sema7A apart from most PGR-responsive genes that are strongly induced during ovulation (Dinh et al., 2023).

### SEMA7A protein localization in apical granulosa cells of antral follicles

**FIGURE 2:**
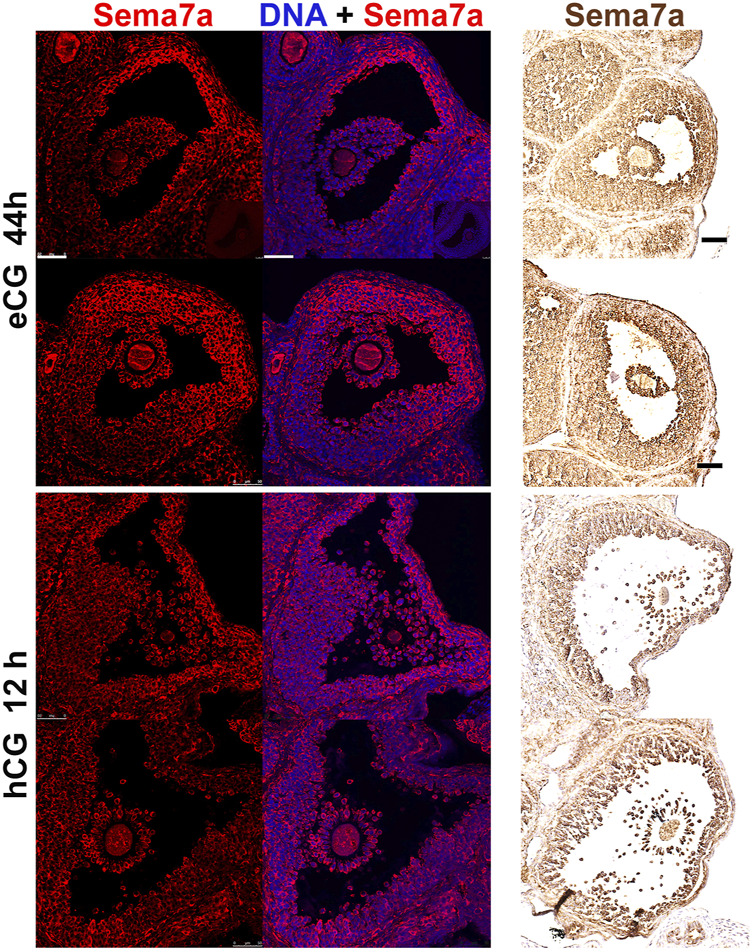
Semaphorin 7A protein localization in apical granulosa cells. Immunofluorescent and immunohistochemical labeling of semaphorin 7A in preovulatory (eCG 44 h treated) or periovulatory (eCG + hCG 12 h) ovaries. All panels are arranged with the follicle apex and ovary exterior to the right hand side of the image. In the immunofluorescent images on the left, semaphorin 7A is labeled in red and DAPI nuclear counterstain in blue. Immunohistochemical labeled images on the right show semaphorin 7A in brown. Scale bars 50 μm.

Immunostaining of SEMA7A protein in antral follicles confirmed its abundance in granulosa cells, predominantly at the apical side of preovulatory follicles (Figure 2). This specific pattern of staining was evident in all large antral follicles imaged and was observed with independent fluorescence or colorimetric immunolabeling methods. Consistent with the downregulation of Sema7A mRNA after hCG administration, the intense apical staining of SEMA7A protein was markedly reduced in sections of ovaries after 12 h hCG treatment.

### Spatiotemporal localization of the SEMA7A adhesion receptor Itga5 during the periovulatory period

**FIGURE 3:**
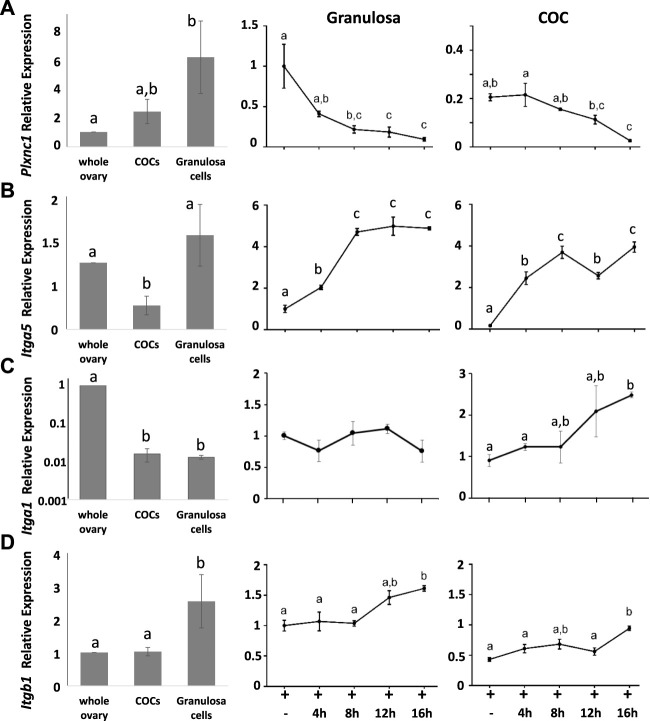
Dynamic switching in semaphorin receptor expression in the periovulatory period. Comparison of **(A)** *PlxnC1*, **(B)** *Itga5*, **(C)** *Itga1*, and **(D)** *Itgb1* expression by RT-qPCR in whole ovaries versus isolated COCs or granulosa cells. Line graphs on RHS show the temporal change in response to hCG ovulation stimulus of each gene in isolated granulosa cells and COCs expressed relative to granulosa cells at 0 h hCG time point.

**FIGURE 4:**
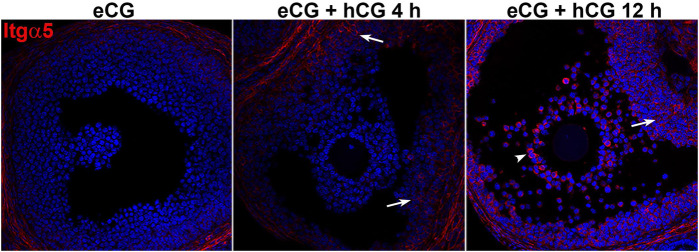
Integrin alpha 5 protein abundance increases during the periovulatory period. Immunofluorescence labeling of integrin alpha 5 (red) in ovarian sections before and 4 or 12 h after ovulation stimulus with hCG. Nuclei are counterstained with DAPI (blue). Arrows indicate positive staining of integrin a5 in granulosa cells 4 and 12 h post-hCG, and arrowheads indicate positively stained COCs 12 h post-hCG.

Semaphorin 7A receptors *Plxnc1*, *Itga1*, *Itga5*, and *Itgb1* were also highly expressed in preovulatory stage ovaries, although less enriched in granulosa and COCs (Figure 3). The Sema7A receptor associated with cell repulsion and retraction, *Plxnc1*, was enriched in granulosa cells and significantly downregulated after ovulation induction, reminiscent of the Sema7A response (>10-fold downregulation post-hCG). The adhesive *Itga5* receptor was significantly increased 5-fold and 20-fold in periovulatory granulosa cells and COCs, respectively (Figure 3). Semaphorin 7A adhesive receptor *Itga1* was expressed 100-fold less in granulosa cells and COCs compared to the whole ovary, and it showed modest regulation by hormones (Figure 3). *Itgb1*, encoding the β-subunit of heterodimeric ITGA1 and ITGA5 Sema7A adhesive receptors (Li et al., 2023), was enriched in granulosa cells with high and relatively stable expression throughout the periovulatory period (Figure 3). The increase in integrin α5 protein in granulosa cells and cumulus cells was confirmed through immunofluorescence. This showed high immunopositive integrin α5 detection in the stroma surrounding follicles, while staining was very low in granulosa and cumulus compartments of preovulatory (eCG-treated) follicles. Integrin α5 abundance within the follicle progressively increased 4 and 12 h after ovulatory stimulation with hCG (Figure 4).

### Concentration-dependent adhesive and repulsive interactions of fibronectin and semaphorin 7A with granulosa cells

**FIGURE 5:**
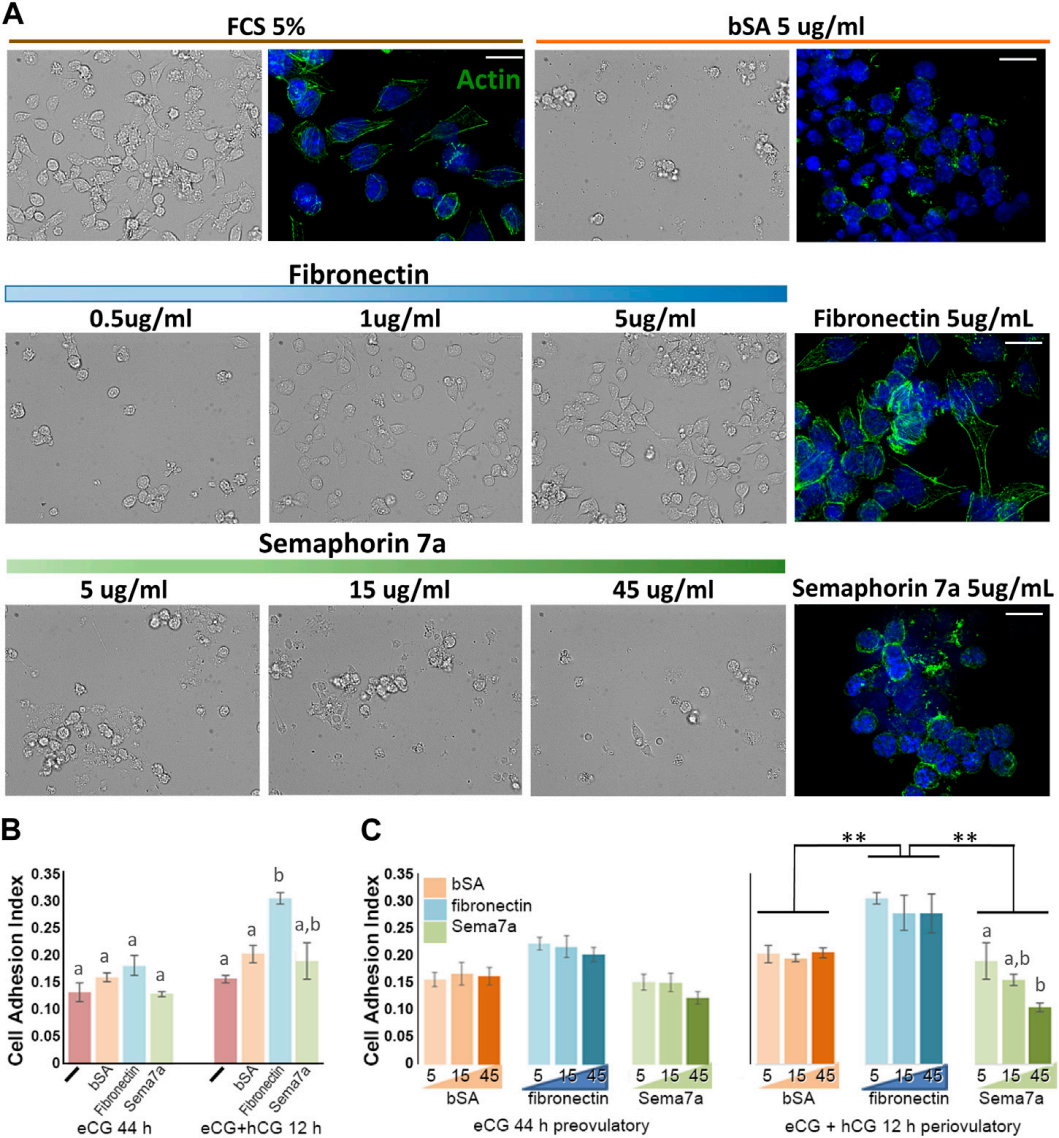
Reciprocal adhesion or repulsion of granulosa cells to fibronectin and semaphorin 7A. **(A)** Bright field and fluorescence images of granulosa cells 2 h after culture on fetal calf serum (FCS), bovine serum albumin (bSA), fibronectin, or semaphorin 7A at the doses indicated. Actin cytoskeleton was labeled using phalloidin-488 (green fluorescence), cell nuclei were labeled usnig DAPI (blue). Scale bars = 15 µm. (**B, C)** Cell adhesion indices recorded 2 h after plating granulosa cells from preovulatory (eCG) or periovulatory (hCG 12 h) follicles, either with no ECM substrate (-) or wells coated with bSA, fibronectin, or semaphorin 7A. **(B)** Comparison of the adhesion index on each ECM in preovulatory and periovulatory granulosa cells. **(C)** Analysis of dose–response relationship of each ECM at the indicated concentrations from 5 to 45 μm. **, significant difference *p* < 0.01. Bars with different letter superscripts are significantly different.

The dynamic temporal regulation by hormones and the spatial pattern of Sema7A and its receptors in ovulating ovarian follicles suggest that they may together participate in the dynamic remodeling of the follicle structure during folliculogenesis and ovulation. The effect of Sema7A on granulosa cells was demonstrated through culture on various ECM substrates (Figure 5). As expected, within 2 h, granulosa cells from eCG-treated mice adhered and established a flattened, elongated morphology, and the actin cytoskeleton formed fine elongated stress fibers in wells coated with fetal calf serum (FCS 5%). In wells coated with non-adhesive bovine serum albumin (bSA 5 μg/mL), cells were rounded, and the actin cytoskeleton coalesced around the nucleus (Figure 5A). Likewise, granulosa cells in fibronectin-coated wells showed low adherent, rounded morphology at 0.5 μg/mL fibronectin but showed increased adherence and flattening in wells with 1–5 μg/mL, and robust actin stress fibers were evident on 5 μg/mL fibronectin substratum. Granulosa cells exposed to Sema7A at concentrations up to 45 μg/mL remained rounded, with a perinuclear actin cytoskeleton in cells coated on 5 μg/mL Sema7A. An increasing concentration of the Sema7A substrate resulted in fewer adherent cells observed, consistent with increased repulsive effects.

Adhesion was quantitatively compared in preovulatory (eCG primed) versus periovulatory (eCG + hCG 12 h) granulosa cells using xCELLigence real-time cell analysis E-plates coated with ECM. In this context, the adhesion index recorded in preovulatory (eCG-primed) granulosa cells with uncoated, or bSA-, fibronectin-, or Sema7A-coated (5 μg/mL) wells was indistinguishable. After hCG 12 h treatment, adhesion to fibronectin was significantly increased (Figure 5B). ECM substrate concentrations from 5 to 45 μg/mL had little impact neither on eCG-primed granulosa cell adhesion nor on the degree of increase in adhesion to fibronectin by hCG-treated periovulatory granulosa cells. However, increasing Sema7A concentrations resulted in the reduced adhesion index with increasing Sema7A concentration in periovulatory granulosa cells (Figure 5C). Again, this is consistent with increased repulsion by Sema7A.

## Discussion

Ovarian follicles are composite assemblies of cells with a range of specialized roles in hormone responsiveness and oocyte maturation. At ovulation, the follicle structure is remodeled to release the matured oocyte through a concerted mechanism controlled by PGR. Our findings show that the semaphorin 7A cell guidance mechanism participates in tissue patterning of the follicle, and control of this mechanism through PGR is a key event in ovulation. Although other semaphorin family members have reported functional roles in ovarian folliculogenesis, particularly Sema4C, Sema4D, and Sema6C support granulosa cell contacts, as well as proliferation and steroidogenic function in preantral follicles (Regev et al., 2007; Yan et al., 2019; Chen et al., 2023), this is the first description of *Sema7A* in ovarian function. Considering the well-known role of semaphorin 7A as guidance cues that regulate cell and ECM contact and tissue organization (Jongbloets et al., 2013), the striking spatial patterning and dynamic hormonal regulation of Sema7A and its partner receptors involved in repulsion or adhesion of cells potentially contributes to the remodeling of the follicle structure during ovulation and luteinization.

Combined actions of FSH and hypoxia promote the expression of Sema7A in granulosa cells of preovulatory follicles. The combined transcriptional control likely establishes the asymmetric spatial pattern, with the semaphorin 7A protein most abundantly localized at the apical follicular regions. It has been demonstrated that the apical region of follicles is more hypoxic (Migone et al., 2016); hence, the spatial pattern of semaphorin 7A is likely established through an O_2_ gradient from the basal vasculature to the apical region of the follicle. This asymmetrical structure of the follicle may be important for controlling the position of COCs and the eventual apical rupture. Upon induction of ovulation, Sema7A expression was rapidly and dramatically reduced, requiring PGR expression. Downregulation of Sema7A is one of a relatively small group of genes that are PGR dependently reduced in the periovulatory period. Our previously reported RNA-seq in PRKO mouse granulosa cells defined 180 genes are PGR dependently induced, and 56 genes (23%) are downregulated (Dinh et al., 2023). We have shown that PGR is a critical transcriptional regulator of ovulation through induction of key PGR-regulated tissue remodeling genes, such as the protease ADAMTS1 that is essential for the COC matrix structure and oocyte release from the follicle (Robker et al., 2000; Brown et al., 2010). Together with the present findings, this indicates that PGR plays a master regulatory role, both inducing and repressing genes to control ovulatory tissue remodeling. We have defined the ovary-specific mechanism of transcriptional activation by PGR that mediates the unique and essential ovulatory transcriptional program (Dinh et al., 2019; Dinh et al., 2023), but PGR-mediated repression is less well understood. The PGR-induced transcriptome includes many secondary transcription factors, including Zbtb16, a well-described transcriptional repressor (Smith et al., 2022); thus, Sema7A downregulation by PGR may involve a secondary transcriptional repressor. The role of a secondary mechanism in PGR-dependent downregulation is also supported since PGR was previously found to promote cyclic increases in Sema7A expression in the hypothalamus via a secondary event involving PGR-induction of Tgfb1 (Parkash et al., 2015).

Tissue organization through semaphorin 7A guidance cues also requires local expression of adhesive or repulsive receptors. In preovulatory granulosa and cumulus cells, high expression of the repulsive receptor *PlxnC1* is downregulated after induction of ovulation, while the adhesive receptor *Itga5* is equally rapidly and highly induced, producing a change in the interactions of granulosa cells and COCs with adjacent cells and ECM in the periovulatory period. We previously showed that expanded periovulatory COCs acquire adhesive, migratory, and invasive capacity on fibronectin and collagen matrices (Akison et al., 2012). Integrin α5, which forms a heterodimer with β1 integrin, promotes adhesion and migration on fibronectin, and the increased *Itga5* expression in COCs provides a mechanistic explanation for that property of COCs. Semaphorin 7A and fibronectin share the RGD tripeptide motif that also binds several integrin dimers, including α5β1 integrin. Here, we found that preovulatory and periovulatory granulosa cells adhere and spread out, forming actin stress fibers indicative of the establishment of focal adhesion contacts on fibronectin but not on semaphorin 7A substrates. Upregulation of *Itga5* mRNA and protein in periovulatory granulosa cells and COCs was consistent with significantly increased adhesion to fibronectin after hCG induction of ovulation. Periovulatory granulosa cells not only increased their adhesive index to fibronectin but also showed dose-dependent repulsion on the semaphorin 7A substratum. This evidence for the repulsive action of semaphorin 7A toward granulosa cells, coupled with its pattern of localization in follicles *in vivo*, suggests that semaphorin 7A may establish a specialized zone, refractory to intercellular interactions at the follicle apex. Reduced Sema7A expression coupled with a switch between *PlxnC1* and *Itga5* expression in response to the ovulatory signal rapidly changes the affinity of intercellular interactions and likely contributes to the remodeling of the follicle structure required for ovulation and formation of the corpus luteum.

In summary, these findings suggest that the regulation of Sema7A and its associated receptors, along with the modulation of integrin α5, contribute to establishing the multilaminar ovarian follicle structure and facilitating the remodeling and apical release of the cumulus–oocyte complex during ovulation. Interestingly, progesterone also mediates cyclic changes in Sema7A in the hypothalamus, mediating cytoskeletal plasticity via inverse responses to PLXNC1 and ITGA1/B1 receptors to facilitate extension and retraction of GnRH neurons at proestrus and diestrus, respectively, and hence, cyclic release of GnRH into the pituitary portal, as well as gonadotrophin release (Parkash et al., 2015). Our findings show that, in turn, gonadotrophins and progesterone/PGR mediate cyclic ovarian remodeling through dynamic regulation of Sema7A and its receptors, changing guidance cues, and, hence, remodeling ovarian follicles, leading to ovulation. Thus, multiple PGR-directed pathways coordinate to mediate oocyte release, and identifying these mechanisms presents new insights and targets for the development of fertility therapies and non-hormonal contraceptives.

## Data Availability

The raw data supporting the conclusion of this article will be made available by the authors, without undue reservation.
